# Pre-vaccination RT-PCR negative contacts in workplace settings show high, SARS COV-2 neutralizing antibody levels

**DOI:** 10.1186/s12889-022-14381-5

**Published:** 2022-10-25

**Authors:** Ridma P Karunathilake, Sameera Hewage, Gihani Vidanapathirana, Athula Kumara, Prabhath Ranasinghe, Faseeha Noordeen, Indika Gawarammana, Champa N Ratnatunga

**Affiliations:** 1 Department of Microbiology, Faculty of Medicine, University of Peradeniya, Peradeniya, 20400 Sri Lanka; 2 Office of the Regional Director of Health Services, Kandy, Sri Lanka; 3 Department of Medical Laboratory Science, Faculty of Allied Health Sciences, University of Peradeniya, Kandy, Sri Lanka; 4 Provincial Department of Health Services, Kandy, Central Province Sri Lanka; 5 Department of Medicine, Faculty of Medicine, University of Peradeniya, Peradeniya, 20400 Sri Lanka

**Keywords:** COVID-19, Workplace settings, PCR negative contacts, Neutralizing antibodies, Risk factors

## Abstract

**Background:**

Asymptomatic SARS-CoV-2 infection occurring in RT-PCR negative individuals represent a poorly characterized cohort with important infection control connotations. While household and community-based studies have evaluated seroprevalence of antibody and transmission dynamics in this group, workplace-based data is currently unavailable.

**Methods:**

A cohort study was carried out in July 2021, during and immediately following the peak of the 3^rd^ wave of COVID-19 in Sri Lanka, prior to mass vaccination. A total of 92 unvaccinated individuals between the ages of 17–65 years were purposively sampled from an office and two factory settings. The selected cohort that had been exposed to RT-PCR positive cases in the workplace was tested RT-PCR negative. Serological samples collected six weeks post exposure were tested for anti-SARS-CoV-2 neutralizing antibody.

**Results:**

The seroprevalence for SARS-CoV-2 specific neutralizing antibodies in the overall cohort was 63.04% (58/92). Seroprevalences in the office setting, factory setting 1 and factory setting 2 were 69.2% (9/13), 55.7% (34/61) and 83.33% (15/18), respectively. Primary risk factor associated with seropositivity was face to face contact with no mask for > 15 min (*p* < 0.024, Odds Ratio (OR); 5.58, 95%CI;1.292– 25.65). Individuals with workspace exposure had significantly higher levels of neutralizing antibodies than those who did not (percentage neutralization in assay 63.3% (SD:21)vs 45.7% (SD:20), *p* = 0.0042), as did individuals who engaged socially without protective measures (62.4 (SD:21.6)% vs 49.7 (SD:21)%, *p* = 0.026).

**Conclusion:**

There was a high seroprevalence for SARS-CoV-2 specific neutralizing antibodies among RT-PCR negative contacts in workplace settings in Sri Lanka. Higher levels of transmission of SARS-CoV-2 infection than estimated based on RT-PCR positive contact data indicate need for targeted infection control measures in these settings during future outbreaks.

**Supplementary Information:**

The online version contains supplementary material available at 10.1186/s12889-022-14381-5.

## Introduction

Beginning in Wuhan, China in December 2019, severe acute respiratory syndrome corona virus-2 (SARS-CoV-2) causing COVID-19, spread across the globe with unprecedented speed, being declared a global pandemic in March 2020 by the WHO [1]. The first wave of COVID-19 in Sri Lanka occurred from March to October 2020 with only 3396 cases, 13 deaths, and a low case fatality rate (CFR) of 0.38, following strict lockdown measures. The second wave, from October 2020 to April 2021 gave rise to 92,341 cases and 591 deaths with a case fatality rate of 0.64. Lockdown measures, case isolation and contact tracing were extensively performed. The third wave starting in April 2021 gave rise to a national health and socio-economic crisis with 445,336 new cases, 13,139 deaths and a high case fatality rate of 2.95, with the delta variant (B.1.617.2) being the dominant viral strain responsible for the high transmission and increased death rates [2, 3]. Restricted movement directives were in place; however, allowance was made for maintenance of agriculture, essential services and export production for economic reasons. While social interaction among the population was minimal during this time, work-based interaction in some sectors continued due to economic needs. As of January 2021 the population seroprevalence of antibodies in the commercial hub of Colombo, where much of the case load was concentrated, was 24.5% [4].

Symptomatic SARS-CoV-2 infection has been the primary focus of global attention. However, asymptomatic infection is important as asymptomatic individuals i) can transmit the infection [5, 6] and ii) affect the estimation of the true disease burden in a community and therefore Infection Fatality Rate (IFR) and basic reproduction number (R0) [7], which indicate how well a community is dealing with an outbreak. Current meta analyses indicate that asymptomatic infection accounts for 20–40% of COVID-19 infection worldwide [8].

Asymptomatic SARS-CoV-2 infection has been shown in both RT-PCR positive [9] and RT-PCR negative individuals [10] the latter confirmed by serological testing, though studies remain sparse. Investigations into serological evidence of infection in RT-PCR negative asymptomatic individuals is sparse [11].

COVID-19 transmission dynamics are affected by environmental factors like indoor/outdoor ventilation, contact patterns (proximity to index case, time, duration and frequency of the exposure) and socio-economic factors (prolonged working hours, indoor crowding, job security) [12]. Scientific investigation evaluating risk behaviors associated with the transmission of SARS-CoV-2 infection has shown face to face contact with the index case to be the dominant independent risk factor [13, 14]. Apart from COVID-19, history reveals that industrial settings carry a high risk of rapid transmission of airborne infectious diseases such as TB [15] and influenza [16] due to risk of close proximity of individuals, high population density, poor ventilation and longer working hours.

We set out to evaluate the seroprevalence of SARS-CoV-2 neutralizing antibodies among a population of RT-PCR negative close contacts of RT-PCR positive individuals in workplace settings in the Kandy district where there were documented outbreaks of COVID-19 during the 3^rd^ wave (starting from May 2021) immediately prior to the national vaccine rollout.

Our objectives were to ascertain the seroprevalence and level of neutralizing antibodies generated in response to asymptomatic RT-PCR negative infection and to evaluate the association of seropositivity and level of neutralization with risk behaviours and risk work environments.

## Methods

### Study population

**Fig. 1:**
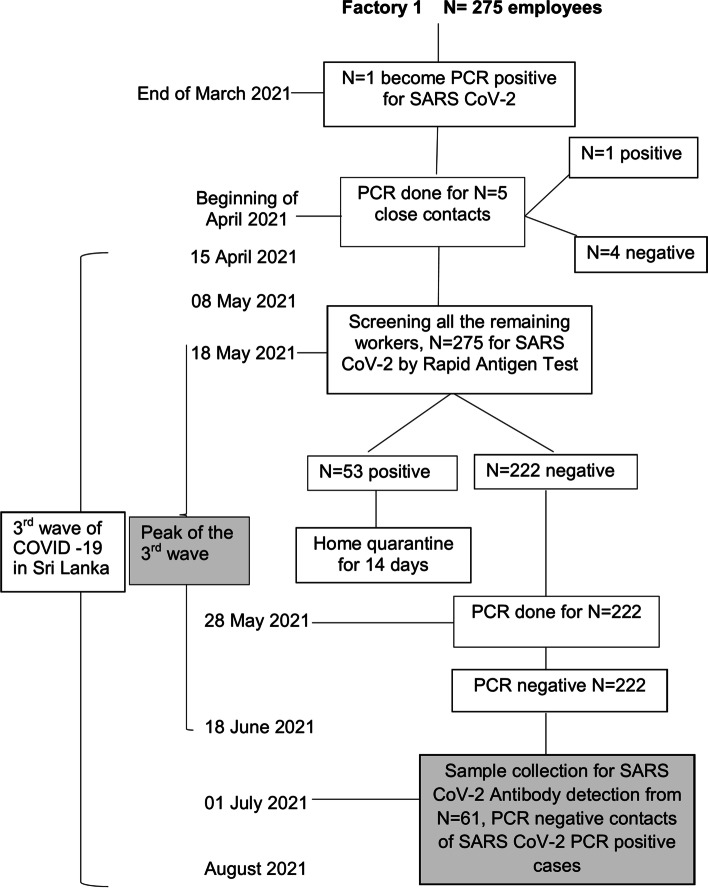
Timeline at Factory 1. The diagram shows the timeline of events related to COVID-19 in Sri Lanka, and the investigation of COVID19 cases and contacts as well as participant recruitment at Factory 1 which is a large-scale food production setting

**Fig. 2:**
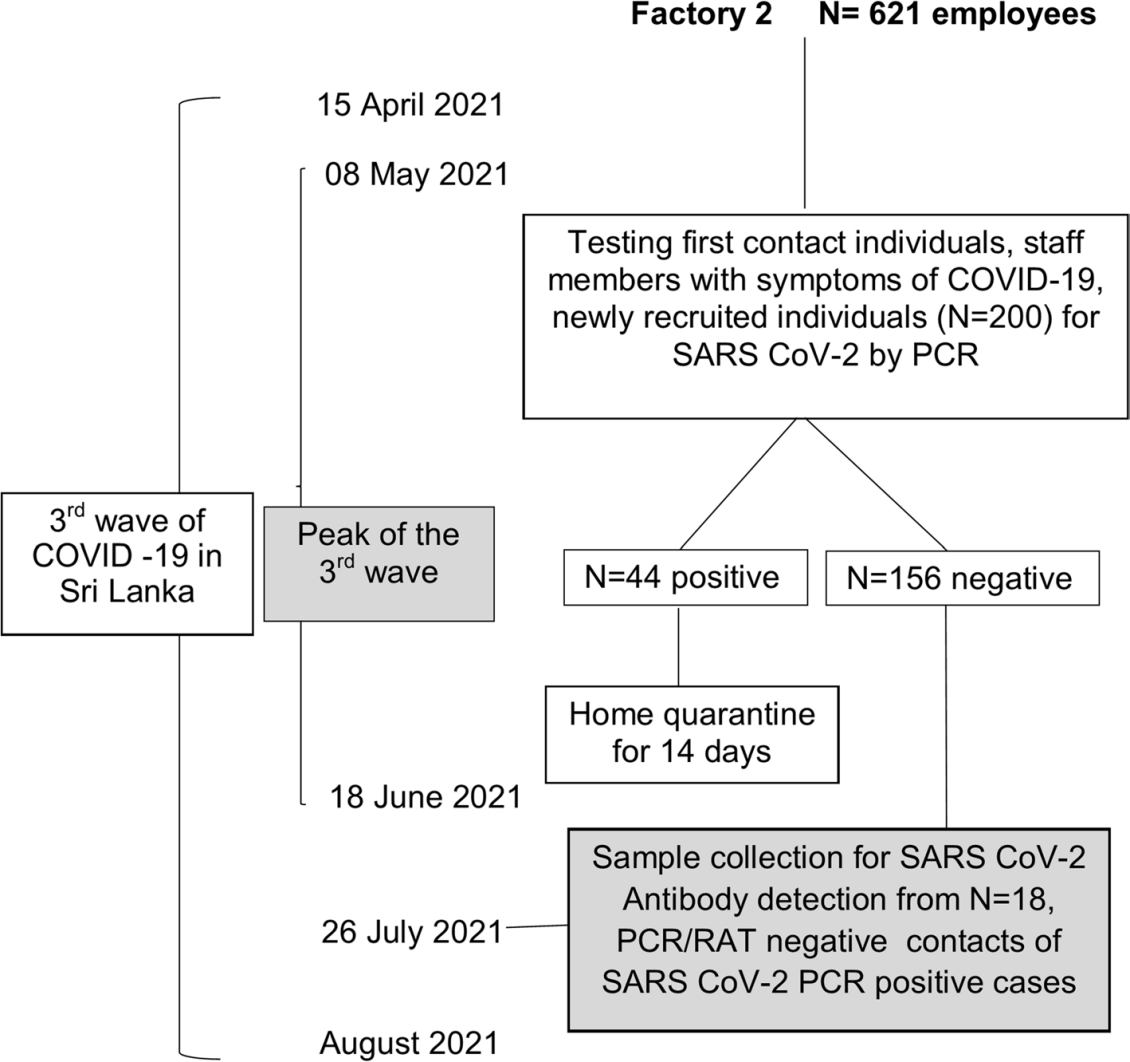
Timeline at Factory 2. The diagram shows the timeline of events related to COVID-19 in Sri Lanka, and the investigation of COVID19 cases and contacts as well as participant recruitment at Factory 2 which is a large-scale garment factory

**Fig. 3:**
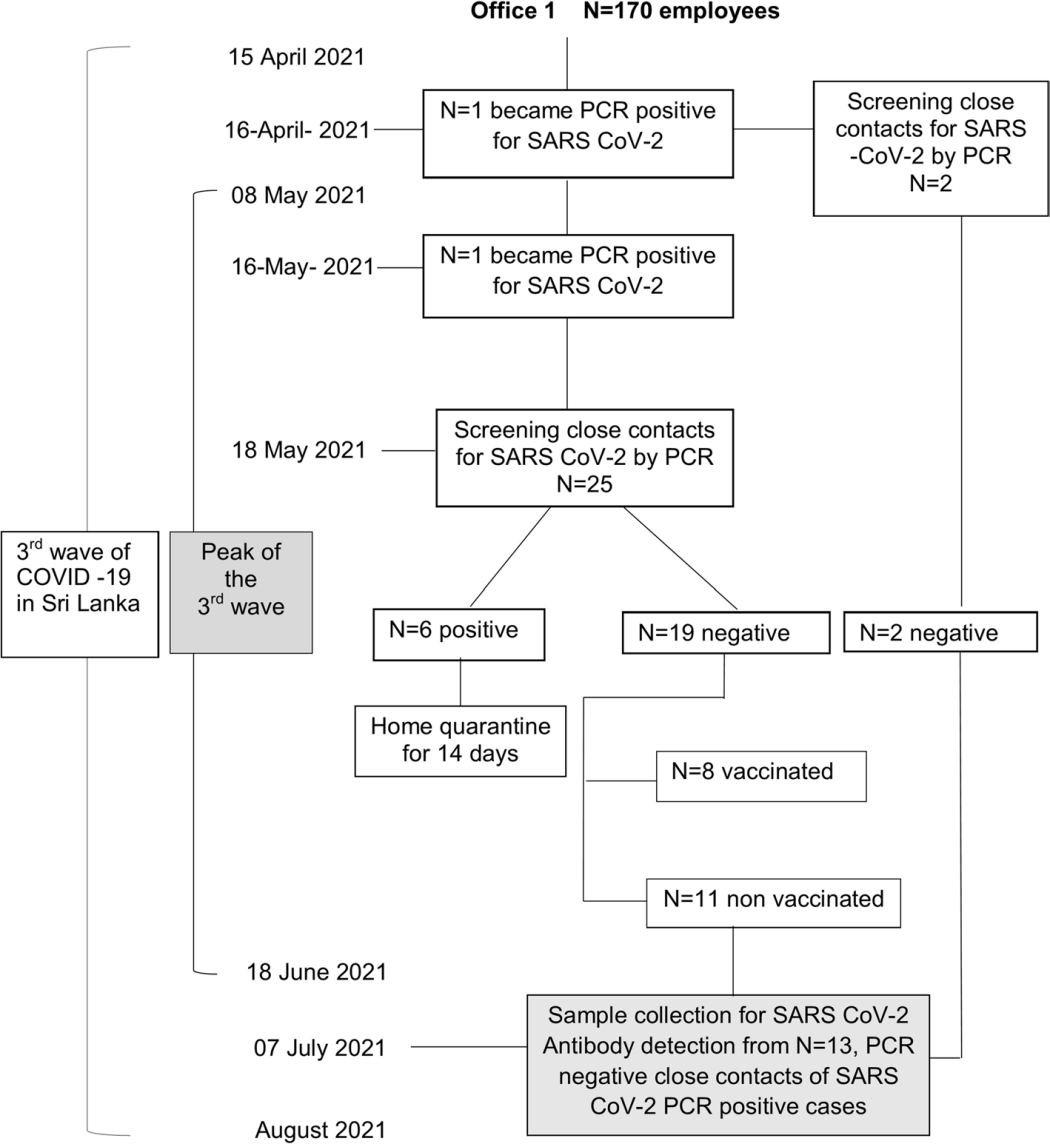
Timeline at Office 1. The diagram shows the timeline of events related to COVID-19 in Sri Lanka, and the investigation of COVID19 cases and contacts as well as participant recruitment at Office 1 which is a medium scale financial sector setting

The study was conducted during July 2021, during and immediately following the peak of the 3^rd^ wave of COVID-19, which was the major outbreak in Sri Lanka, immediately before the national COVID-19 vaccine roll-out. A total of 92 unvaccinated individuals between the ages of 17–65 years working in three institutions; two factory settings (factory-1 and factory-2) and an office setting (office-1) were purposively recruited for the study with informed written consent. The selected individuals had been exposed to RT-PCR positive individuals in the workplace and had been screened during routine community-based, government mandated screening, for COVID-19 at the appropriate time following exposure by RT-PCR and confirmed as negative. RT-PCR for all samples had been performed at the National Hospital Kandy Virology laboratory on nasopharyngeal swabs collected as part of the national community screening and diagnostic programme run by the Provincial and Regional Directorate of Health Services. RT-PCR was performed using the RealStar SARS-CoV RT-PCR kit which used gene targets E gene (βCoV specific) and S gene (SARS CoV specific) for diagnosis. Samples with both target gene amplification (Ct < 40) with valid controls were considered positive. Samples with both target genes negative with valid controls, were considered negative. Blood sample collection for serological testing for this study was done approximately 4–6 weeks from known exposure to detect maximum antibody responses. Figure 1, 2 and 3 show the timeline of events and screening dates at the three selected locations in relation to the ongoing wave of COVID-19 at the time.

Briefly, in factory -1, a medium sized food production factory with 275 employees, an initial exposure event occurred at the end of March 2021 with detection of a single case. Initial screening of five close contacts in April 2021 revealed an additional case. On May 8^th^, as case numbers were increasing with the peak of the 3^rd^ wave of infection in the country, all remaining employees were screened by rapid antigen testing revealing an additional 53 cases. The 222 rapid antigen negative individuals were screened by RT-PCR and ‘confirmed’ as negative. A convenience sample of 61 individuals from this cohort were sampled for antibody testing at the beginning of July 2021.

In factory -2, a medium sized garment factory with 621 employees, 200 individuals were tested by RT-PCR in early May 2021 as they were either symptomatic, first contacts of PCR positive cases at home, or newly recruited. Of those tested 44 were found to be positive while 156 were found to be negative. Individuals with negative RT-PCR were followed up and screened with either repeat RT-PCR or rapid antigen testing if they developed symptoms over the next 2 weeks. A convenience sample of 18 individuals who had remained asymptomatic and had no history of a positive antigen or RT-PCR test, were sampled for antibody testing at the end of July, 2021.

In office setting-1, the third test site with a total of 170 employees, two employees were diagnosed as RT-PCR positive in mid-April and mid-May respectively. Screening of two contacts of the initial case done in April gave no further positives while screening of 25 close contacts of the 2^nd^case done on the 18^th^ of May 2021, revealed 6 cases and 19 negative individuals. Of the 19 negative individuals, 8 were vaccinated and 11 were not. Blood samples from the initial two negative contacts and the latter 11 (total 13) unvaccinated contacts were collected for antibody detection at the beginning of July 2021.

All the participants were asymptomatic at the time of sample collection and had no history of being RT-PCR positive or had any history of COVID-19 suggestive symptoms during the preceding months. Basic demographic details such as age, gender and the date of sample collection for RT-PCR testing for SARS-CoV-2 and detailed exposure history (working in the same workspace for > 2–3 h with and without ventilation, working in the same air conditioned space for > 2–3 h with no other ventilation, face to face contact with no mask for > 15 min, eat/socialize together with no protective measures, travel together in enclosed space < 30 min, travel together in enclosed space > 30 min, share items at meals/clothing/other) were recorded. A follow-up telephone interview was conducted to cross check details filled in the questionnaire including interview of 35 RT-PCR positive individuals from the same settings to cross evaluate the accuracy of responses given by RT-PCR negative individuals.

Blood samples were obtained to detect the presence of neutralizing antibodies against SARS-CoV-2 using the Genscriptc Pass™ SARS-CoV-2 neutralization Antibody Detection kit (GenScript USA/ Nanjing GenScript Diagnostics Technology Co., Ltd., Version RUO for US.1.0). Separated serum samples were stored at -80 °C until testing. Testing was carried out according to manufacturer’s instructions. Percentage inhibition was obtained using the surrogate viral neutralization assay [17]. A cutoff of 30% neutralization (% inhibition) was taken as positive (seroconversion) as recommended by manufacturer. Level of antibody expressed as a percentage neutralization was calculated for each sample.

### Statistical analysis

Statistical analysis was carried out using GraphPad Prism (8.4.2. 679, 2020). Percentage neutralization was compared using Mann Whitney test (due to non-normal distribution of values), and association between categorical variables was evaluated using the chi-square test. Multiple groups were compared with the Kruskal Wallis test and Dunns Post-hoc test for multiple comparisons. Forward and backward stepwise logistic regression analysis was performed to determine independent risk factors for SARS-CoV-2 transmission [18].

## Results

### Demographic characteristics

Demographic characteristics of the cohort are shown in Table 1.

**Table 1:** Demographic characteristics of the study cohort

Characteristic	Value/ Frequency
Gender	
Male	23 (25%)
Female	69 (75%)
Age	
Mean age	40.43 years
Age range	17–65 years
Age SD	10.28
Age categories	
≤ 20	2 (2.17%)
21–30	18 (19.56%)
31–40	20 (21.74%)
41–50	38 (41.30%)
> 50	14 (15.2%)
Educational level	
Passed grade 8	21(22.83%)
Passed GCE^a^ Ordinary Level	47 (51.08%)
Passed GCE^a^ Advanced Level	22 (23.92%)
Completed higher education	2 (2.17%)

^a^*GCE* General Certificate of Education

### Seroprevalence of SARS-CoV-2 specific neutralizing antibodies

**Fig. 4:**
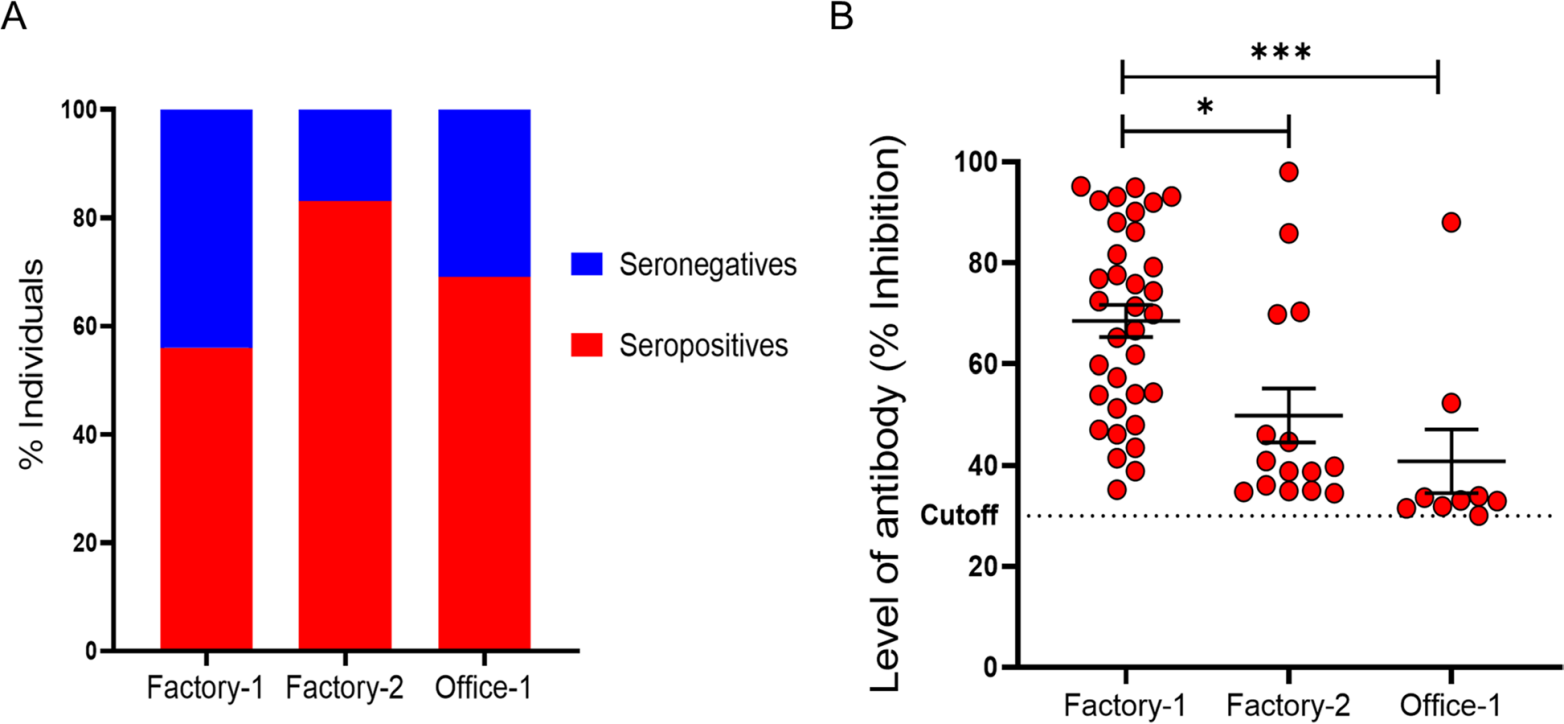
Seroprevalence and level of neutralizing antibodies in seropositive individuals at each setting. Factory-1, Factory-2 and Office-1 were investigated for neutralizing antibodies against SARS CoV-2. **A** shows the seroprevalence for neutralizing antibodies against SARS CoV-2 by study site. **B** shows the neutralizing antibody level expressed as a percentage inhibition in seropositive individuals, by study site. Seropositivity designated at > 30% inhibition in assay (manufacturers standard). Comparison between groups, Kruskal Wallis test, with Dunns post hoc test. *p < 0.01, **p < 0.01, ***p < 0.001

The seroprevalence of SARS-CoV-2 specific neutralizing antibodies in the overall cohort was 63.04% (58/92). Seroprevalence in factory-1 was 55.7 (34/61), in factory-2 was 83.33% (15/18) and in office-1 was 69.2% (9/13). The mean antibody level ( inhibition) of seropositives was 40.8% (95% CI: 26.25–55.36) in office 1, 68.50% (95% CI: 62.02–74.97) in factory 1 and 49.8% (95% CI:38.40–61.34) in factory 2. A comparison of the level of neutralizing antibody (percentage inhibition of assay) in each setting is shown in Fig. 4

Comparison of mean antibody level of the seropositive individuals between study sites revealed a significantly higher level of antibodies (Kruskall Wallis test, *p* < 0.0001) in individuals at factory -1 compared to both factory-2 (Dunns test, *p* = 0.01) and office-1 (Dunns test, *p* < 0.0001). In addition, seropositive individuals from factory-2 had a higher level of neutralizing antibodies compared to office-1 though this did not reach statistical significance. These results reveal that despite having a lower overall seroprevalence than factory-2 and office-1, factory-1 showed the highest levels of neutralizing antibodies between the three institutions while both factory settings had individuals with higher levels of neutralizing antibodies compared to the office setting (Fig. 4).

We then analyzed the behavioral trends associated with seropositivity to ascertain which behavior patterns were associated with seropositivity in these settings.

### Seropositivity and risk behaviours

The primary behavior associated with seropositivity in univariate analysis was having face to face contact with no mask for > 15 min (chi square test df_2_, *p* < 0.024). There was no association between seropositivity and level of workspace exposure (evaluated based on number of hours and ventilation during exposure), exposure during eating/socializing together with no protective measures or exposure by sharing items at meals/clothing/other with RT-PCR positive individuals. As both factories provided transport to and from the workplace for employees, exposure during travel was also evaluated for association with seropositivity, however, gave no significant result. Binary logistic modelling gave a similar result with no further significant risk factors identified. Odds ratios with 95% confidence intervals for each of the evaluated risk factor are given in Table 2.

**Table 2:** Odds ratios with 95% confidence intervals for each of the evaluated risk factor

Risk factor	Odds Ratio	95% Confidence interval	*P* value
Workspace exposure	1.11	0.35– 2.59	> 0.99
Travel exposure	0.59	0.72– 4.06	0.28
Face to face contact with no mask for > 15 min	**5.58**	**1.29– 25.65**	*0.02*
Eat/socialize together with no protective measures	0.54	0.19– 1.71	0.42
Share items at meals/clothing/other	1.93	0.69- 5.58	0.31

**Fig. 5:**
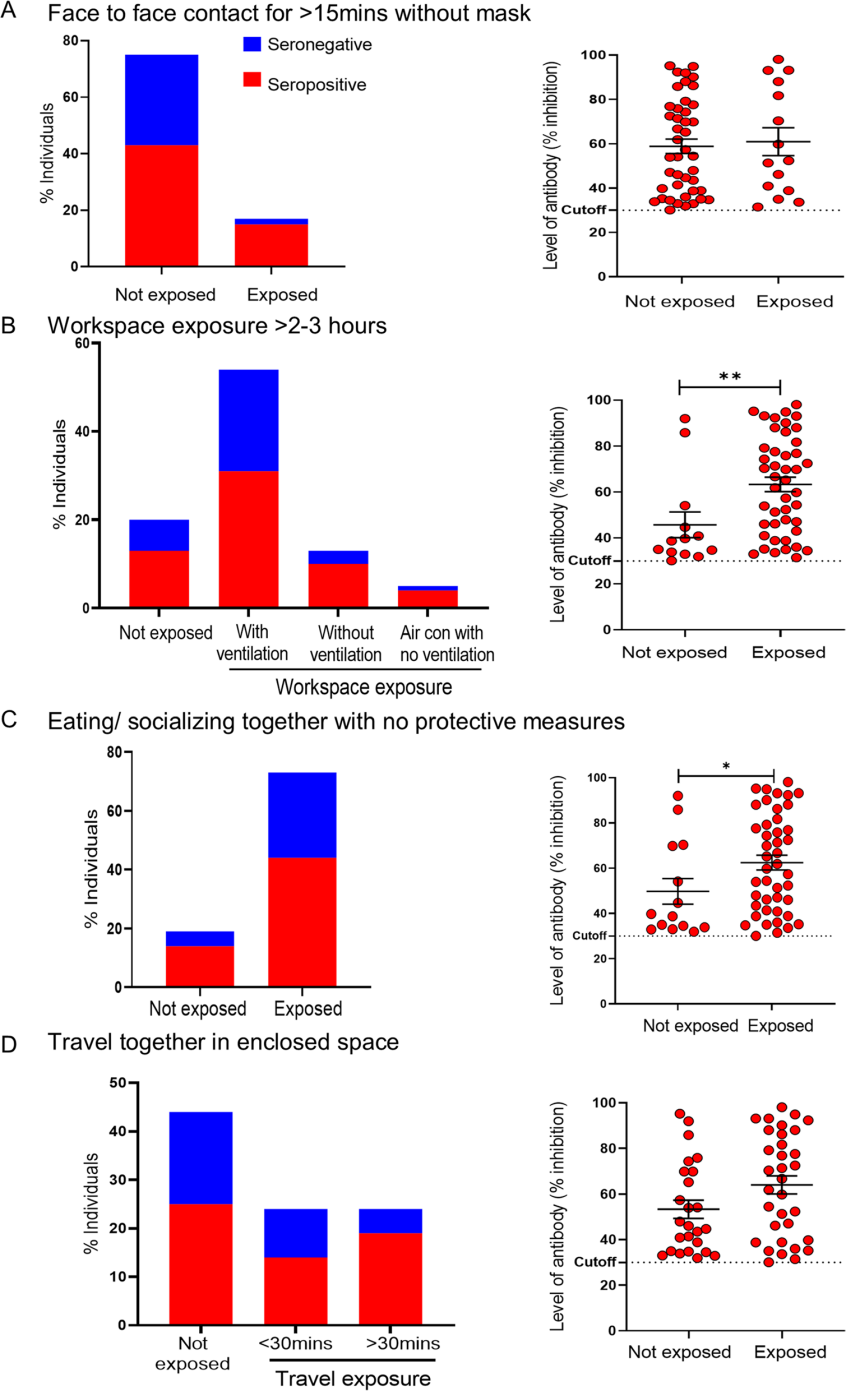
Seropositivity and level of antibody in seropositive individuals by exposure factors. Bar graphs show the percentage of seropositive and seronegative individuals within each exposure category. Comparison between groups, Chi square for trend/ Fisher’s exact test. Dot plots show the level of neutralizing antibody in seropositive individuals as percentage inhibition within each exposure category. Seropositivity designated at > 30% (manufacturers standard). **A** Face to face contact for > 15 min without mask with known RTP-PCR positive individual. **B** Workspace exposure: working in the same environment/ space as known RT-PCR positive individual. **C** Eating or socializing together with known RT-PCR positive individual with no protective measures. **D** Traveling together in company provided transport with known RT-PCR positive individual. Comparison between groups, Mann–Whitney test. *p < 0.05, **p < 0.01, ***p < 0.001

A deeper analysis of seropositive individuals in each of exposure category is shown in Fig. 5. Although face to face contact for > 15 min with no mask was a significant risk factor for seropositivity, there was no significant difference in the level of neutralizing antibody between the seropositive individuals who had such exposure (60.9% (SD:24.3)) and those who did not (58.8%, SD:21.4) (Fig. 5A). In contrast, seropositive individuals with workspace exposure (same workspace for 2–3 h with ventilation, without ventilation or in air conditioned rooms with no other ventilation) had significantly higher neutralizing antibody levels compared to those who had no work place exposure (63.3% (SD:21) vs 45.7(SD:20.2)%, Mann Whitney test, *p* = 0.0042) (Fig. 5B). Similarly, individuals who were seropositive had engaged socially (eating/ socializing together with known COVID-19 positive individuals) without protective measures and these individuals had significantly higher neutralizing antibody levels compared to those who did not engage socially (62.4% (SD:21.6) vs 49.7% (SD:21), Mann Whitney test, *p* = 0.026) (Fig. 5C). Traveling in enclosed space showed a similar trend with those who did so having a mean neutralizing antibody level of 63.9% (SD:22.7) while those who did not travel in enclosed spaces had a mean level of 53.3% (SD:19.8) though this difference did not reach statistical significance (Mann Whitney, *p* = 0.063) (Fig. 5D).

**Fig. 6:**
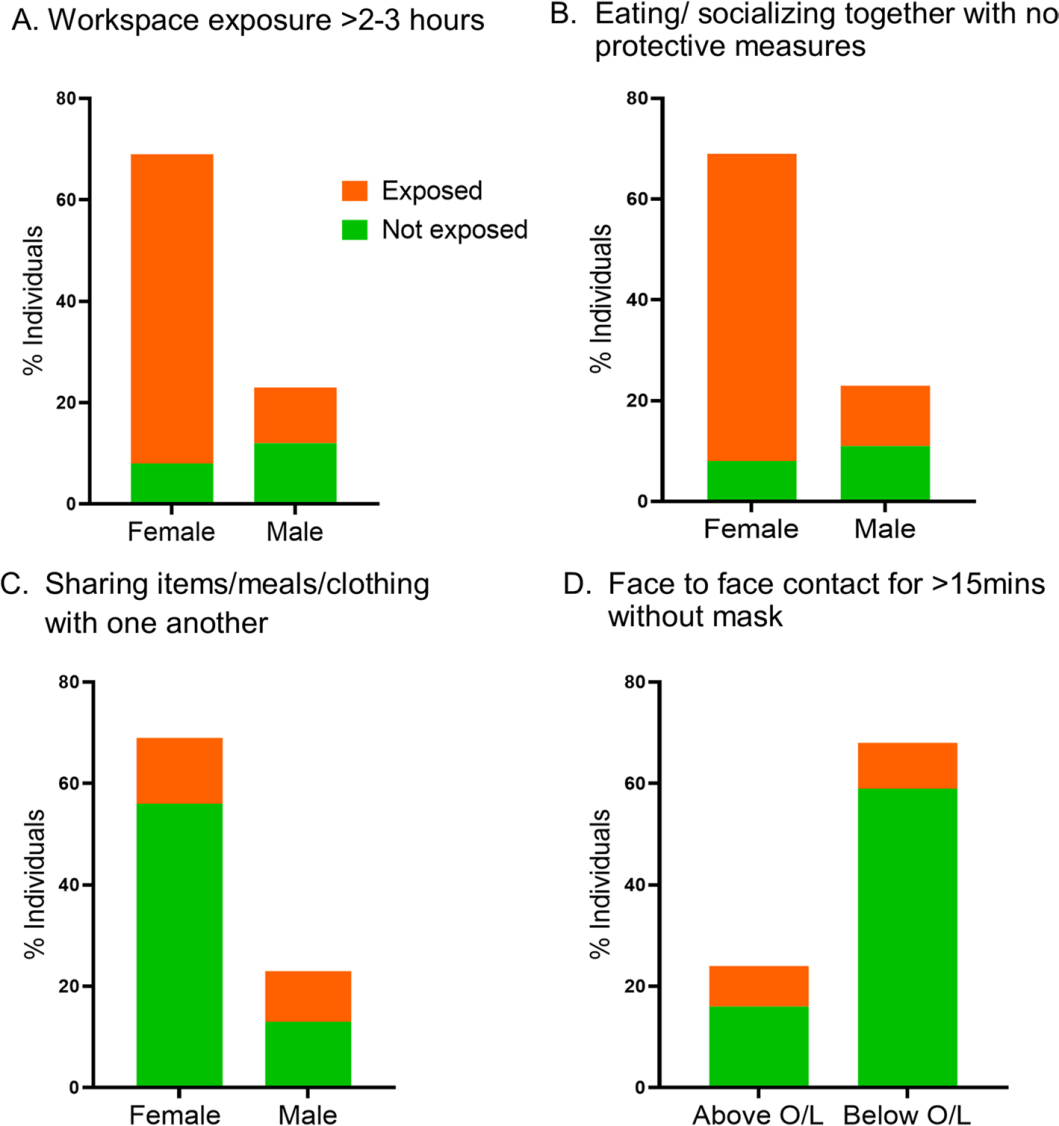
Demographic characteristics of individuals by risk behaviors. Females were more likely to have some degree of workspace exposure to a positive case during working hours compared to males (chi sq test, *p* < 0.001, OR 8.318, 95% CI 2.891–22.87) (**A**) and were significantly more likely to eat/socialize together with no protective measures (*p* < 0.001, OR 6.99, 95% CI 2.389–19.05) (**B**). In contrast, males were more likely to share items/meals/clothing/ with one another (*p* = 0.018, OR 3.314, 95% CI 1.245–8.66) (**C**) in the study cohort. Moreover, having an education level of Ordinary Level examination or above was associated with being more likely to have face to face contact with no mask for > 15 min (*p* = 0.029, OR 3.287,95% CI 1.074–10.28) (**D**)

Having identified risk behaviors/ factors associated with seropositivity, we investigated the demographic characteristics of those who engaged in risk behaviors (Fig. 6.).

### Validity of responses

A telephone interview carried out including 35 SARS-CoV-2 RT-PCR positive workers from the factory setting who were index cases of the study cohort revealed that some responses regarding exposure history given by the RT-PCR negative study cohort may not be reliable. For example, only 25% (23/92) of the RT-PCR negative group responded that they had shared items of food during meals and / or clothing while 88.57% (31/35) of the RT-PCR positive group said otherwise. Moreover, only 5.43% (5/92) of RT-PCR negative group claimed to have worked in same air-conditioned space for > 2–3 h with no other ventilation whereas 40% (14/35) in the RT-PCR positive group stated the opposite. A significant difference was observed between the contradictory responses given by the two groups regarding sharing items during meals/clothing/other (*p* < 0.001, OR = 23.25, 95% CI-7.273 to 64.62) and working in the same air-conditioned space (*p* < 0.001, OR = 11.6,95% CI -2.295 to 5.970).

Overall, we show that while > 15 min of face-to-face contact without mask was the primary risk factor for being seropositive in this cohort, seropositive individuals had higher antibody levels if they had high risk workspace exposure (> 2–3 h in the same space) or engaged socially with RT-PCR positive individuals without protective measures. Telephone interviews with RT-PCR positive employees revealed that the number of those engaging in risk behavior in the contact group may be underestimated.

## Discussion

We demonstrate a high seroprevalence for SARS-CoV-2 specific neutralizing antibodies in SARS-CoV-2 RT-PCR negative contact population in three workplace settings in the Kandy district of Sri Lanka during the third wave of COVID-19 (July 2021) immediately prior to vaccine roll-out. This is the first data from industrial/ office settings available globally to the best of our knowledge. The workplace seroprevalence of 63.04% is markedly higher than the community and household seroprevalence data shown in other studies worldwide. A community based study by Ng et al. carried out between Jan 23 and April 3, 2020 in Singapore on close contacts who were either PCR negative or asymptomatic and untested gave a 3.8% seropositivity [14] while a study from China conducted between 20 January and 29 February 2020 on PCR negative household contacts gave a 23.6% seroprevalence [19]. Weis S. et al. reported a 5.1% seroprevalence in PCR negative close contacts from 12^th^ to 22^nd^ May, 2020 in a quarantined village in Germany 6 weeks following a COVID-19 outbreak. [20]. These present the few data sets available on antibody seroprevalence in PCR negative study cohorts and are early data sets that are household/ community based. The seroprevalence, which included both RT-PCR positive and negative individuals, in the general population of densely populated Colombo in January 2021 was shown to be 24.5% which is < 50% of that demonstrated in our study cohort, which comprised workers primarily from semi-urban and rural areas of the Kandy district. Our results highlight the significant transmission of asymptomatic infection occurring in workplace settings compared to community settings, particularly in densely populated factory settings. Meta analysis of overall seroprevalence (not limited to asymptomatic/ RT-PCR negative individuals) across 968 studies which included 74 countries with 202 prevalence data showed that though general population seroprevalence was approximately 4.5%, a wide variation existed in specific populations, with a high 59% prevalence in persons in assisted living and long term care facilities [21]. Seroprevalence was also higher in people aged 18–64 compared to over 65 (RR1.27, 95% CI 1.11–1.45). These findings indicate that higher seroprevalence can be expected in working age individuals in high population density settings consistent with virus transmission dynamics. Given that our data represents seroprevalence at a later (mid 2021) stage of the pandemic in workplace settings where deficiencies in infection control would have occurred, the seroprevalence we have found appears reasonable, though disturbingly high. In addition to seroprevalence we show that neutralizing antibody levels are also higher in the densely populated factory settings compared to the office setting indicating that the level of exposure may have an impact on the strength of the humoral immune response even in asymptomatic infection.

To the best of our knowledge, this is the only study to evaluate the level of neutralizing antibodies in RT-PCR negative contacts. Therefore, comparison of these results in either community or workplace settings is not possible. However, comparison with results from two studies carried out in Sri Lanka, utilizing the same surrogate viral neutralization assay in symptomatic and vaccinated populations show that, the mean antibody level in our study cohort (mean antibody level:59.38% 95%, CI: 53.61- 65.16) was similar to that reached with symptomatic infection prior to vaccination (median antibody level: 63.2%, IQR: 9.9- 95.2%) [22] or with single dose vaccination (median antibody level: 69.42%, IQR: 54.09- 81.54% [23] though the statistical significance of these results cannot be tested at this time. These findings suggest that exposure, particularly exposure in confined settings like factory 1 (mean antibody level: 68.50% (95% CI: 62.02–74.97), can result in significant immune responses, despite being both asymptomatic and RT-PCR negative.

Among evaluated risk factors, the only identified risk factor for seropositivity was having face to face contact with no mask for > 15 min. Sample size limitation may have impacted these findings. A study carried out in China demonstrated that transmission among household cases was associated with exposure to multiple cases, face to face communication with an index case for 30 min or longer and sharing a vehicle with an index case. However, no independent association was observed between SARS-CoV-2 transmission and either indirect contact, meal sharing, and lavatory co-usage [14]. A similar study carried out in South Korea showed face-to-face conversations, eating together, and using the same toilet with COVID-19 patient, were significantly associated with the SARS-CoV-2 infection and sharing objects with SARS-CoV-2 infected persons was not associated with acquiring the infection. Moreover, face-to-face conversations with a COVID-19 infected individual was revealed to be an independent risk factor for SARS-CoV-2 transmission (OR: 4.11) [13]. These studies strongly corroborate our findings. In addition, we show that although the evaluated risk factors of workspace exposure, and exposure without protective measures during socializing/ eating did not reach statistical significance for simple seropositivity, such exposure showed higher levels of neutralizing antibodies. These findings also support the likelihood that increased level of exposure is associated with increased humoral antibody response even in asymptomatic infection. This is the first-time neutralizing antibody level has been evaluated in relation to exposure risk factors. This evidence strongly supports the recommendations to reduce COVID-19 transmission, presented by the Three Cs Strategy of WHO (avoid i. crowded places ii. close-contact settings iii. confined and enclosed spaces) and the CDC, which encourage the public to wear face masks, maintain physical distance from other persons, limit in-person contacts, avoid non-essential indoor spaces, protect essential workers by providing adequate personal protective equipment, follow safe work practices and to increase air ventilation and environmental disinfection in the workspace [24].

Male: female ratio in office-1, factory-1 and factory-2 were 9:4, 8:53 and 1:2 respectively. Demographic characteristics such as gender and education level were found to be associated with increasing the risk behavior for potential exposure for COVID-19. Females were more likely to eat and socialize together with no protective measures while males were tended to share items at meals/clothing/other. More educated population who had completed their studies beyond GCE O/L seemed to be sharing items/ meals/clothing/other and maintaining face to face contact with no mask for > 15 min than the less educated population. These results highlight the socio-cultural differences in habits that need to be specifically addressed if consistent changes in behavior are to be brought about and that possibly health literacy is more important than formal education in preventing COVID-19. Gender based differences in attitudes towards pandemic restrictions have been consistently shown over 8 countries with females being more likely to agree and comply with restrictions on behavior [25]. Here we show that certain behavior patterns in some communal settings do not conform to largescale evaluations. Investigation of ingrained etiquette in a given setting and addressing these at a ground level are likely to be necessary for good infection control practice in individual institutions.

There was significant disparity between the responses given by PCR positive and negative groups regarding the exposure history. It is possible that the significantly higher likelihood of risk behavior seen in the PCR positive group was the reason they became infected. However, it is also possible and indeed more likely that the PCR negative participants were hesitant to reveal their true exposure history, possibly due to fear of repercussions from the workplace administration for not following guidelines issued by the government to reduce the transmission of SARS-CoV-2. Moreover, the level of stigma at that point of time in the society and not having psychological safety to reveal the truth may have discouraged them from revealing pertinent facts related to their exposure history. Therefore, underestimation of risk behavior is likely. This is another area that needs to be addressed when improving infection control by creating a non-threatening environment, which empowers employees to understand and report risk behaviors.

The primary strength of this study is that it is the only data currently available showing evidence of transmission in RT-PCR negative contacts in workplace settings. In addition to seropositivity, the added dimension of level of neutralizing antibodies and the association of exposure factors to both give new insights into the relationship between exposure and induced immunity. The main weakness it the small sample size due to practical restrictions, which limited the number of tests we could perform at each study site. Increasing both the number of samples as well as study sites would have provided a clearer picture of transmission dynamics in different workplace settings,

Collectively our findings indicate high levels of SARS-CoV-2 transmission occur in workplaces, and these individuals are not included in the total case count of a country or region unless seroprevalence studies are performed. This has several implications. First, the total number of COVID-19 cases in Sri Lanka is likely to be much higher than current estimates. The corollary of which is that the true case fatality rate for Sri Lanka during the 3^rd^ wave is likely lower than 2.55%, which is above the global average (1.7%). Second, while workplaces, particularly industrial settings were kept functional during the lockdowns to maintain economic solvency, there was inadequate preparation of the workplaces and the employees for infection control. Third, the subsequent vaccine rollout that covered the working population has resulted in higher levels of immunity than just the two-dose regime would give as many workers in industrial settings are likely to already have seroconverted despite negative PCR results. The subsequent 4^th^ wave of infection by the delta and omicron variants and the current 3^rd^ dose deployment island wide mean that fairly high antibody levels can be expected in this population. This is good news for subsequent waves with new variants as high levels of at least partially neutralizing antibodies can be expected in the workforce.

Further work exploring the adequacy of infection control measures and adherence to country and company policy in ground level workplaces is needed especially to flatten the curve until the effect of vaccine role out is gained. COVID-19 will not be the last pandemic we face. The need of developing economies running without being damaged by prolonged lockdowns highlights the need for policy and structural changes that ensure continuous functioning of workplaces.

## Conclusion

There was a high seroprevalence for SARS-CoV-2 specific neutralizing antibodies in the workplace setting in Sri Lanka in asymptomatic, RT-PCR negative contacts. True SARS-CoV-2 infection rates are underestimated and transmission and control of infection in workplace settings is an area that requires specific attention.

## Supplementary Information

**Additional file 1.** **Supplemantary data table 1**: Optical density values and % inhibition values of the serum samples collected from the workplace settings.

## Data Availability

ELISA data are analyzed and presented in this study are available in supplementary data Table 1. Demographic, socio-economic and risk behavior data are available from the corresponding author on reasonable request.

## Abbreviations

CDC: Centers for Disease Control and Prevention
CI: Confidence Interval
COVID-19: Corona Virus Disease 2019
IFR: Infection Fatality Rate
OR: Odds Ratio
PCR: Polymerase Chain Reaction
RT-PCR: Real Time Polymerase Chain Reaction
SARS-CoV-2: Severe Acute Respiratory Syndrome Coronavirus 2
SD: Standard Deviation
TB: Tuberculosis
WHO: World Health Organization
